# Impacto en la salud pública del sistema de telediagnóstico implementado en Paraguay

**DOI:** 10.26633/RPSP.2017.74

**Published:** 2017-04-14

**Authors:** Pedro Galván, Miguel Velázquez, Gualberto Benítez, José Ortellado, Ronald Rivas, Antonio Barrios, Enrique Hilario

**Affiliations:** 1 Departamento de Ingeniería Biomédica e Imágenes Instituto de Investigaciones en Ciencias de la Salud, Universidad Nacional de Asunción Paraguay Departamento de Ingeniería Biomédica e Imágenes, Instituto de Investigaciones en Ciencias de la Salud, Universidad Nacional de Asunción, Paraguay.; 2 Ministerio de Salud Pública y Bienestar Social Ministerio de Salud Pública y Bienestar Social Asunción Paraguay Ministerio de Salud Pública y Bienestar Social, Asunción, Paraguay.; 3 Universidad del País Vasco Universidad del País Vasco Bilbao España Universidad del País Vasco, Bilbao, España.

**Keywords:** Salud pública, tecnología biomédica, telemedicina, ingeniería sanitaria, radiología, telerradiología, tecnología de la información, Paraguay, Public health, biomedical technology, telemedicine, sanitary engineering, radiology, teleradiology, information technology, Paraguay, Saúde pública, tecnologia biomédical, telemedicina, engenharia sanitária, radiologia, telerradiologia, tecnologia da informação, Paraguai

## Abstract

**Objetivo.:**

Determinar la viabilidad y puesta en marcha de un sistema de telediagnóstico para dar asistencia sanitaria a poblaciones remotas y dispersas del Paraguay.

**Métodos.:**

El estudio fue realizado en todos los hospitales regionales, generales y principales hospitales distritales de las 18 regiones sanitarias del Paraguay. En el sistema se registraron los datos clínicos y las imágenes tomográficas, ecográficas y trazados electrocardiográficos del paciente que precisaba de un diagnóstico por parte de un médico especialista. Esta información se transmitió a los especialistas en imagenología y en cardiología para su diagnóstico remoto y posterior envío del informe a los hospitales conectados al sistema. Se analizó el costo-beneficio e impacto de la herramienta de telediagnóstico desde la perspectiva del Sistema Nacional de Salud.

**Resultados.:**

Entre enero de 2014 y mayo de 2015 se realizaron 34 096 telediagnósticos distribuidos en 25 hospitales a través del Sistema de Telemedicina del Ministerio de Salud. El costo unitario promedio del diagnóstico remoto fue de USD 2,6 (dólares estadounidenses) para electrocardiograma (ECG), tomografía y ecografía, mientras que el costo unitario para el diagnóstico “cara a cara” fue de UDS 11,8 para ECG; USD 68,6 para tomografía y USD 21,5 para ecografía. La reducción del costo mediante el diagnóstico remoto fue de 4,5 veces para ECG; 26,4 veces para tomografía y de 8,3 veces para ecografía. En términos monetarios, la implementación del sistema de telediagnóstico, durante los 16 meses del estudio, significó un ahorro promedio de USD 2 420 037.

**Conclusión.:**

*Paraguay cuenta con un sistema de telediagnóstico para electrocardiografía, tomografía y ecografía aplicando las tecnologías de la información y comunicación (TIC) de bajo costo, basadas en* software *libre y escalable a otros tipos de estudios diagnósticos a distancia; de interés para la salud pública. Con una aplicación práctica del telediagnóstico, se contribuyó al fortalecimiento de la red integrada de servicios y programas de salud, lo que permitió maximizar el tiempo del profesional y su productividad, mejorar la calidad, aumentar el acceso y la equidad, y disminuir los costos.*

## Abstract (en)

**Objective.:**

Determine the viability of a remote diagnosis system implemented to provide health care to remote and scattered populations in Paraguay.

**Methods.:**

The study was conducted in all regional and general hospitals in Paraguay, and in the main district hospitals in the country’s 18 health regions. Clinical data, tomographic images, sonography, and electrocardiograms (ECGs) of patients who needed a diagnosis by a specialized physician were entered into the system. This information was sent to specialists in diagnostic imaging and in cardiology for remote diagnosis and the report was then forwarded to the hospitals connected to the system. The cost-benefit and impact of the remote diagnosis tool was analyzed from the perspective of the National Health System.

**Results.:**

Between January 2014 and May 2015, a total of 34 096 remote diagnoses were made in 25 hospitals in the Ministry of Health’s telemedicine system. The average unit cost of remote diagnosis was US$2.6 per ECG, tomography, and sonography, while the unit cost of “face-to-face” diagnosis was US$11.8 per ECG, US$68.6 per tomography, and US$21.5 per sonography. As a result of remote diagnosis, unit costs were 4.5 times lower for ECGs; 26.4 times lower for tomography, and 8.3 times lower for sonography. In monetary terms, implementation of the remote diagnosis system during the 16 months of the study led to average savings of US$2 420 037.

**Conclusion.:**

Paraguay has a remote diagnosis system for electrocardiography, tomography, and sonography, using low-cost information and communications technologies (ICTs) based on free software that is scalable to other types of remote diagnostic studies of interest for public health. Implementation of remote diagnosis helped to strengthen the integrated network of health services and programs, enabling professionals to optimize their time and productivity, while improving quality, increasing access and equity, and reducing costs.

## RESUMO

**Objetivo.:**

Avaliar a viabilidade e a implementação de um sistema de telediagnóstico destinado a oferecer assistência de saúde a populações remotas e dispersas do Paraguai.

**Métodos.:**

O estudo foi realizado em todos os hospitais regionais e gerais e nos principais hospitais distritais das 18 regiões sanitárias do Paraguai. Foram registrados no sistema os dados clínicos, as imagens tomográficas e ecográficas e os traçados eletrocardiográficos de pacientes que precisavam de um diagnóstico por parte de um médico especialista. Estas informações foram transmitidas a especialistas em diagnóstico por imagem e cardiologia para que fizessem o diagnóstico remoto e enviassem então os laudos aos hospitais conectados ao sistema. Analisou-se a relação custo-benefício e o impacto da ferramenta de telediagnóstico da perspectiva do Sistema Nacional de Saúde.

**Resultados.:**

Entre janeiro de 2014 e maio de 2015, foram realizados 34.096 telediagnósticos em 25 hospitais através do Sistema de Telemedicina do Ministério da Saúde. O custo unitário médio do diagnóstico remoto foi de US$ 2,6 (dólares americanos) para eletrocardiografia (ECG), tomografia e ecografia, enquanto que o custo unitário para o diagnóstico presencial foi de US$ 11,8 para ECG, US$ 68,6 para tomografia e US$ 21,5 para ecografia. A redução do custo pelo uso do diagnóstico remoto foi de 4,5 vezes para ECG, 26,4 vezes para tomografia e 8,3 vezes para ecografia. Em termos monetários, a implementação do sistema de telediagnóstico, ao longo dos 16 meses do estudo, representou uma economia média de US$ 2.420.037.

**Conclusão.:**

O Paraguai conta com um sistema de telediagnóstico para eletrocardiografia, tomografia e ecografia que utiliza tecnologias da informação e comunicação (TIC) de baixo custo, baseadas em *software* livre e ampliáveis a outros tipos de exames diagnósticos à distância que são de interesse para a saúde pública. A aplicação prática do telediagnóstico contribuiu para o fortalecimento da rede integrada de serviços e programas de saúde, o que permitiu maximizar o tempo dos profissionais e sua produtividade, melhorar a qualidade, aumentar o acesso e a equidade e reduzir os custos.

La telemedicina en su significado más amplio consiste en prestar asistencia sanitaria a distancia (remota). Por su parte, el telediagnóstico consiste básicamente en la parte que se ocupa del diagnóstico. El sistema de telediagnóstico incorpora herramientas de las tecnologías de la información y comunicación (TIC), en especial de la web y bases de datos.

La telemedicina aplicada en la atención sanitaria permite atender a pacientes que viven alejados de los hospitales de la capital y sin especialistas (1, 2). En nuestro caso, consideramos que nos puede permitir planificar en función del perfil epidemiológico del país y ofrecer una cobertura universal en áreas diagnósticas de referencia en el nivel nacional tan importantes como son la electrocardiografía (ECG), la tomografía y la ecografía, así como impulsar un modelo de salud pública basada en la equidad y universalidad (Declaración de Alma Ata de las Naciones Unidas) (3), sin descuidar la utilidad de las tecnologías involucradas. Así, las TIC ofrecen importantes posibilidades para mejorar la cobertura de los servicios e intercambiar con mayor efectividad informaciones clínicas, administrativas, de capacitación del personal y de socialización de la información científica con la población afectada (4).

A pesar de que las TIC son muy prometedoras, existen pocos estudios que avalen de manera rotunda la idoneidad, el costo-beneficio y el impacto socioeconómico para solucionar problemas concretos en determinadas áreas geográficas (5–9). En tal sentido, para conocer la posibilidad de implementar de manera sistemática el uso de la telemedicina en el Paraguay, el Departamento de Ingeniería Biomédica e Imágenes del Instituto de Investigaciones en Ciencias de la Salud (IICS) de la Universidad Nacional de Asunción (UNA) realizó en 1999, junto con el Ministerio de Salud Pública y Bienestar Social (MSPBS), una prueba piloto vía satélite en un servicio de teleecografía (10), cuyos resultados no fueron muy prometedores. Esto se debió a que la tecnología vía satélite no era sustencuadro en el sector público por el alto costo del ancho de banda necesario. A partir del año 2007, se comenzó a trabajar con el actual Sistema de Telemedicina, gracias a una importante mejora del servicio de internet en el IICS y al aumento de la conectividad de las instituciones y de la población en general.

Para ello, la Unidad de Telemedicina del MSPBS, en colaboración con el Departamento de Ingeniería Biomédica del IICS-UNA, y con el apoyo técnico de la Universidad del País Vasco, Bilbao (España), realizó un estudio para evaluar el costo-beneficio e impacto socioeconómico del sistema de telemedicina en la salud pública. Este estudio supuso una importante fuente de información objetiva e independiente sobre la viabilidad técnica para implementar y sustentar la ejecución de proyectos de telemedicina para diagnóstico y consultas de especialistas a distancia en los centros asistenciales del Paraguay.

## MATERIALES Y MÉTODOS

### Período de estudio

El estudio se realizó entre enero de 2014 y mayo de 2015.

### Selección de los hospitales

Se identificaron los hospitales regionales, distritales y generales, de las 18 regiones sanitarias en las que se divide el país y que no contaban con servicios diagnósticos de electrocardiografía, tomografía (sin contraste) y ecografía ginecoobstétrica.

Se instaló la aplicación para el servicio de telediagnóstico en 25 hospitales en el nivel nacional. En 24 de ellos se instaló el servicio de teleelectrocardiografía (tele-ECG), en seis se instaló teletomografía y, en cuatro, teleecografía.

### Población estudiada

El estudio incluyó 34 096 pacientes con solicitud médica de estudios diagnósticos por imagen (tomografía y ecografía) y señales eléctricas biológicas (ECG) que acudieron durante el período de estudio a los 25 hospitales citados. Los datos de los pacientes fueron consignados en una ficha electrónica.

### Equipamiento y *software* utilizados

En el caso del ecógrafo, se utilizó una tarjeta de captura para acceder a la señal de video análogo y luego poder ser transferido al ordenador mediante un cable de S-video. Con el tomógrafo se utilizó un ordenador específico donde se descargaron las imágenes digitales en formato DICOM para luego procesarlas y almacenarlas a través de un *software* propietario. Para los estudios electrocardiográficos se dispuso de una conexión RS-232 que, a través del puerto COM, permitió interactuar con el ordenador mediante un *software* de aplicación, lo que nos facilitó la captura de la información y la posterior generación de gráficos en formato .jpg. La aplicación web fue utilizada por los especialistas en imagenología médica y electrocardiografía para así simplificar el proceso de incorporación de las imágenes obtenidas por los respectivos equipos periféricos de diagnóstico a la base de datos de la ficha electrónica del paciente.

La tecnología digital utilizada para la transmisión de las imágenes en este estudio se denomina *store & forward*. Una vez obtenidas las imágenes por el técnico sanitario, se ejecutó el módulo de ficha electrónica del paciente (aplicación *stand alone* o web) a través de determinado protocolo. El “especialista remoto” (profesional médico especialista en imagenología, ecografía o cardiología) visualizó, al entrar en el sistema, los datos clínicos de los pacientes y las imágenes anexas para poder realizar el diagnóstico, el cual estaba de manera inmediata disponible para su impresión y entrega al paciente y para su envío por correo electrónico al médico del hospital remoto.

Las imágenes captadas y procesadas fueron remitidas al médico especialista vía internet. El muestreo fue no probabilístico de conveniencia. Para asegurar la confidencialidad de la información así como su integridad y consistencia se han utilizado mecanismos tales como el acceso controlado al sistema (usuario y contraseña), consultas priorizadas por tipo de usuario (secretaría, técnico, médico o administrador del sistema), bases de datos codificadas, comunicación codificada tipo *secure sockets layer* (SSL) y llaves de codificación para la manipulación y modificación de la información mediante el uso de un protocolo de encriptación que provee comunicación segura.

### Análisis de costo-beneficio e impacto

El análisis de costo-beneficio permitió comparar los costos incurridos por las dos alternativas (diagnóstico “a distancia” versus “cara a cara” en un hospital de Asunción) para determinar el beneficio, que se mide en unidades monetarias.

Para calcular el beneficio del sistema de telediagnóstico en el caso del diagnóstico remoto se consideraron los costos de implantación, el mantenimiento de las TIC y del informe médico. Para el diagnóstico “cara a cara” con el especialista médico en Asunción se consideraron los costos de transporte, la manutención, la oportunidad y el arancel médico. Es decir, se buscó el costo-efectividad o, dicho de otra manera, el coste de la inversión de los recursos disponibles frente a la mejor inversión y alternativa disponible. Los costos referenciales utilizados en el análisis para el diagnóstico remoto se extrajeron de los contratos entre el Ministerio de Salud y los profesionales imagenólogos, cardiólogos y ecografístas del sistema de telediagnóstico. En el caso del diagnóstico “cara a cara” fueron extraídos de los aranceles vigentes establecidos por las sociedades paraguayas de cardiología, radiología y ecografía para Asunción. También se incluyeron en los costes del sistema “cara a cara” los costos de transporte, manutención y oportunidad para el viaje desde el interior del país a Asunción. Para el cálculo del coste de transporte, se utilizó la tarifa económica establecida para el viaje en transporte público convencional por la Dirección Nacional de Transporte. El coste de oportunidad es el coste por cada día laboral perdido durante el viaje (ingreso caído) para realizar el estudio en la capital. Para el cálculo del coste de alimentación y oportunidad se utilizó el valor del jornal mínimo diario establecido por el Estado paraguayo (Ministerio de Hacienda 2014/15) para un obrero o jornalero de poca cualificación, como es el caso de la gran mayoría de usuarios de los servicios de telediagnóstico en el interior del país.

La base de datos del sistema de telediagnóstico estaba disponible para toda la red de servicios de salud del MSPBS (Dirección de Telemedicina, médicos especialistas informantes, hospitales remotos de la red), con restricciones de acceso mediante contraseña según el nivel jerárquico del que accede.

### Evaluación del impacto económico-social

Para evaluar el impacto y poder medir los efectos que se podrían generar con la implantación del sistema de telediagnóstico, se procedió a identificar, valorar y determinar los cambios que ha provocado la introducción del diagnóstico a distancia en la práctica económica, social y del conocimiento de los 25 hospitales del sistema nacional de salud.

En cuanto a los indicadores del impacto social en términos de salud se ha valorado la relación causa-efecto que existe entre la implantación del telediagnóstico con la accesibilidad al diagnóstico especializado, adecuado y protocolizado en las áreas de electrocardiografía, tomografía y ecografía en los 25 hospitales estudiados y sus áreas de influencia.

Se estima que los indicadores del impacto en términos de salud se corresponden con los efectos directos que se obtienen con la utilización del sistema de telediagnóstico sobre la salud del paciente. En este sentido, se valoraron midiendo la disminución de la morbilidad y mortalidad de los casos materno-fetales, cardiológicos y traumatológicos, así como el incremento del índice de supervivencia de los pacientes diagnosticados en forma temprana y adecuada. El impacto económico directo nos viene dado por la obtención de beneficios económicos directos a los pacientes afectados por los tres servicios de telediagnóstico.

### Evaluación del diagnóstico clínico

Para la evaluación del diagnóstico clínico se consideraron los siguientes indicadores implícitos: variabilidad de la prescripción del estudio, tecnología del equipo, aplicación, transmisión de imágenes y experiencia en la interpretación de las mismas. Para la evaluación de los criterios anteriores, la medida común en que se expresó la efectividad del telediagnóstico fue el porcentaje de “diagnósticos correctos” en comparación con el diagnóstico convencional realizado por el especialista “cara a cara”. Esta evaluación fue realizada por médicos expertos que analizaron la concordancia del diagnóstico remoto frente al diagnóstico “cara a cara”. El trazado e imagen primaria generada por el equipo de ECG, ecógrafo y tomógrafo constituyó el patrón de oro para determinar el diagnóstico. El diagnóstico realizado con la imagen o trazado primarios fue contrastado con el diagnóstico realizado con las imágenes y trazados bajados de la plataforma del sistema de telemedicina vía internet.

### Encuesta de satisfacción

Por último, una encuesta de satisfacción semiestructurada en línea de usuarios se aplicó a 7 de los 10 médicos especialistas (70%) en telediagnóstico (5 médicos imagenólogos y 5 médicos cardiólogos) y a 39 de los 50 técnicos sanitarios (78%) (técnicos en radiología, enfermeras y obstetras), que utilizaron la herramienta del sistema de telediagnóstico. La escala de satisfacción fue: 1: no estoy de acuerdo en nada; 2: estoy en desacuerdo; 3: me es indiferente; 4: estoy de acuerdo; 5: estoy muy de acuerdo.

El presente estudio contó con la aprobación del Comité de Ética y se realizó respetando las consideraciones relativas al cuidado de los participantes en investigación clínica incluidas en la Declaración de Helsinki y con acuerdo a la Guía para Investigaciones en Salud Humana (Resolución 1480/11) del Ministerio de Salud de la Nación Argentina y del Ministerio de Salud de Paraguay. Este estudio solo utilizó las imágenes y la información clínica de forma anónima, y no presentó ningún tipo de riesgo para los participantes ni para su confidencialidad. Toda información obtenida fue utilizada por los investigadores con la más estricta confidencialidad solo para los fines del presente estudio.

Debido a la naturaleza retrospectiva y anónima del estudio, no se solicitó consentimiento informado, ya que por un lado las unidades de observación fueron los lugares donde se llevaron a cabo los estudios y, por otro lado, se procedió de tal manera que no existió ninguna manera de vincular los estudios con las personas reales que originaron las imágenes.

## RESULTADOS

De los 34 096 telediagnósticos realizados a través del sistema de telemedicina de la Dirección de Telemedicina del MSPBS, el 41% correspondieron a varones y el 59% a mujeres. Representaron el total de casos diagnosticados a distancia con historias clínicas ajustadas al propósito de la investigación. La edad media de los pacientes fue de 48 años.

**FIGURA 1.**
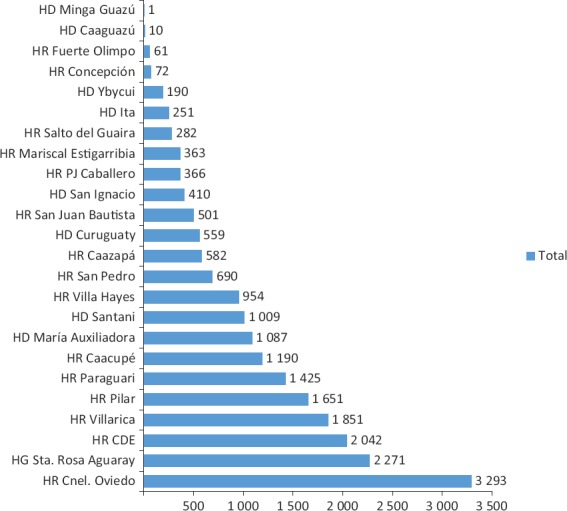
Total de electrocardiogramas de las 24 localidades.

**FIGURA 2.**
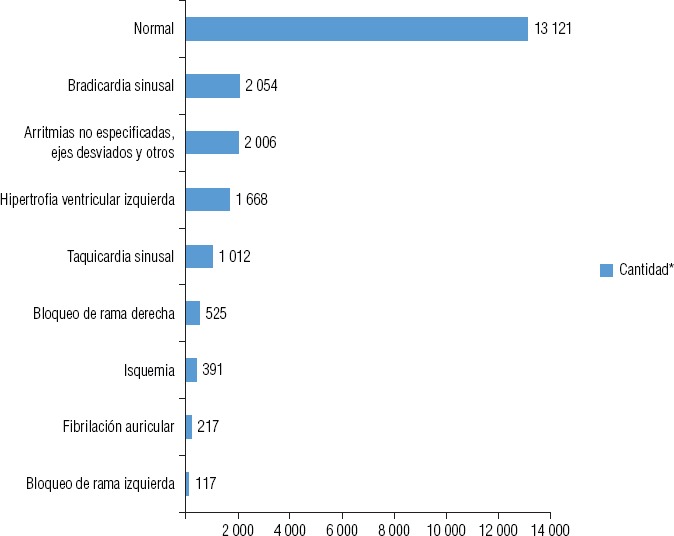
Tipo y cantidad de estudios electrocardiográficos (n = 21 111).

El mayor número de diagnósticos a distancia fueron electrocardiográficos, con un total de 21 111, que correspondieron en su gran mayoría a chequeos médicos de rutina. La distribución de los estudios electrocardiográficos realizados a distancia por hospital comunitario se muestra en la figura 1. En cuanto a los casos diagnosticados en los hospitales del interior del país, los resultados arrojaron los siguientes diagnósticos: normal (62,2%), bradicardia sinusal (9,7%), arritmias no especificadas (ejes desviados, trastornos de la conducción, escasa progresión de onda R y secuelas de infarto) (9,5%), hipertrofia ventricular izquierda (7,9%), taquicardia sinusal (4,8%), bloqueo de rama derecha (2,5%), isquemia (1,9%), fibrilación auricular (1%) y bloqueo de rama izquierda (0,5%). La distribución del tipo y cantidad de diagnósticos de ECG realizados puede observarse en la figura 2.

**FIGURA 3.**
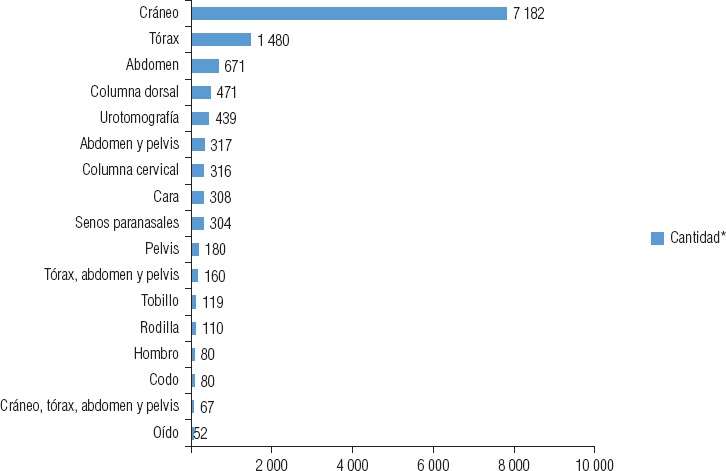
Tipo y cantidad de estudios tomográficos (n = 12 966).

La mayoría (60,2%) de los 12 966 estudios de tomografía fueron de la región de la cabeza, como consecuencia del gran número accidentes de tráfico. La distribución del tipo y cantidad de estudios tomográficos realizados puede observarse en la figura 3.

Los 19 estudios de ecografía a distancia correspondieron a controles prenatales del área de ginecoobstetricia. De ellos, 14 se realizaron en Fuerte Olimpo, tres en Mariscal Estigarribia, uno en Villa Hayes y uno en Pilar. La razón de la escasa cantidad de diagnósticos de ecografía se debió a la reticencia de los profesionales ecografístas, quienes preferían el diganóstico con la imagen impresa en papel. Resulta evidente que hace falta una mayor sensibilización entre los médicos ecografístas para el uso costo-efectivo de la herramienta.

### Análisis de costo-beneficio

En el análisis de los resultados obtenidos de los costos promedios de diagnóstico remoto se consideraron solamente los costos de aquellos hospitales que realizaron más de 125 estudios de ECG, 51 de tomografía y tres de ecografía. En el caso de los estudios de ECG y de tomografía correspondían a valores umbrales o de inflexión, a partir de los cuales el telediagnóstico se vuelve rencuadro en comparación al diagnóstico “cara a cara”. En el caso de ecografía se incluyó el costo promedio de diagnóstico remoto a partir de tres estudios, ya que el valor umbral o de inflexión de 100 estudios aun no fue alcanzado por ninguno de los cuatro hospitales incluidos en este estudio durante el corto período de funcionamiento pleno del sistema. En términos generales, se observó una diferencia importante en el coste de diagnóstico remoto en relación al diagnóstico “cara a cara” (cuadro 1).

**CUADRO 1.** Matriz de cálculo para el análisis del costo-beneficio del sistema de telediagnóstico

Tipo de diagnóstico	Número total de estudios realizados (1)	Costo unitario del informe remoto (2) (USD/estudio)	Costo promedio del diagnóstico remoto (3) (USD/estudio)	Costo unitario del informe cara a cara (4) (USD/estudio)	Costo promedio del diagnóstico cara a cara (5) (USD/estudio)	Beneficio del tele-diagnóstico (5–3) x (1) = (6) (USD)
Electrocardiografía	21 111	2,6	10,9	11,8	60,1	1 038 661,2
Tomografía	12 966	2,6	12,6	68,6	120,4	1 397 734,8
Ecografía	19	2,6	974,9	21,5	113,9	16 359,0
Total general	34 096					2 420 037,0

Cuadro de elaboración propia a partir de los resultados presentados.

El costo unitario promedio del informe remoto fue de USD 2,6 USD para ECG, tomografía y ecografía, mientras que el costo unitario promedio para el diagnóstico “cara a cara” fue de 11,8 USD para ECG, 68,6 para tomografía y 21,5 para ecografía. Así pues, la reducción del costo unitario mediante el diagnóstico remoto fue de 4,5 veces para ECG, 26,4 veces para tomografía y de 8,3 veces para ecografía. Esto supone un beneficio importante para cada ciudadano del interior del país, toda vez que el costo promedio de diagnóstico remoto para cada hospital sea igual o inferior al coste total del diagnóstico “cara a cara” (sin contar el desembolso económico que supone acudir a la ciudad costo que tendría que abonar el paciente y acompañantes de “su propio bolsillo”). En términos monetarios, la implementación del sistema de telediagnóstico durante los 16 meses del proyecto piloto en los 25 hospitales significó un ahorro promedio de 2 420 037 dólares americanos. Los diferentes montos promedios ahorrados por tipo de estudio remoto se muestran en el cuadro 1.

### Análisis y evaluación del impacto económico-social

En cuanto a los indicadores del impacto social en términos de salud, de forma directa benefició a 1 497 725 habitantes de las 25 comunidades donde se localizaban los hospitales, y en forma indirecta a 6 503 976 habitantes de las regiones sanitarias del área de influencia de los hospitales con sistema de telediagnóstico, lo que representó una cobertura del 92,7% de la población total del país; según la proyección poblacional de la Dirección General de Estadísticas, Encuestas y Censos del Paraguay.

Hemos podido constatar que el beneficio social ha incluido también el proceso clínico, la salud del paciente y la accesibilidad a la atención sanitaria, que son fortalecidas por el sistema de telemedicina implantado.

En cuanto al impacto económico directo a los pacientes afectados por los tres servicios de telediagnóstico, en el caso de ECG representó un ahorro real de USD 1 038 661,2 y de USD 1 397 734,8 en tomografía, mientras que la ecografía no solo no supuso un ahorro, sino un sobrecosto de USD 16 359. La distribución del impacto social por área del servicio de telediagnóstico se muestra en el cuadro 2.

**CUADRO 2.** Matriz del impacto socioeconómico del sistema de telediagnóstico

Tipo de diagnóstico	Número total de estudios realizados	Número de hospitales con servicio diagnóstico remoto	Población beneficiada en forma directa (ciudad) por el telediagnóstico (habitantes)	Población beneficiada en forma indirecta (región) por el telediagnóstico (habitantes)	Beneficio económico del telediagnóstico (USD)
Electrocardiografía	21 111	24	1 385 135	6 503 976	1 038 661,2
Tomografía	12 966	6	658 771	2 384 515	1 397 734,8
Ecografía	19	4	111 525	273 416	16 359,0
Total general	34 096	25	1 497 725	6 503 976	2 420 037,0

Cuadro de elaboración propia, a partir de los resultados presentados.

### Evaluación del diagnóstico clínico

Al comparar la calidad de las imágenes de pacientes generadas en la pantalla de los equipos médicos de diagnóstico y las disponibles en el sistema de telediagnóstico no se encontraron discrepancias significativas en lo referente a la calidad global de la imagen, aberraciones ópticas y fidelidad. Sin embargo, se determinó que la vulnerabilidad de la conectividad es alta y dependiente de la tecnología de comunicación disponible, que en este caso fue principalmente inalámbrica. La mejor efectividad se logró con los estudios de ECG con 98,3% (n = 21 111). El porcentaje de diagnóstico correcto en el caso de la tomografía fue de 96,1% (n = 12 966) y para ecografía del 84,2% (n = 19). El porcentaje promedio general de diagnósticos correctos y tratamientos adecuados fue del 92,9% (n = 34 096) y el de error en el diagnóstico fue del 7,1%. Los estudios con errores (7,1%), lógicamente, se repitieron.

### Satisfacción sobre la asistencia recibida

El grado de satisfacción expresado por los pacientes en lo referente a la asistencia recibida por parte de los especialistas remoto fue del 94,6%. Además, todos los encuestados manifestaron estar entre “de acuerdo” y “muy de acuerdo” (máxima calificación) con la calidad de la asistencia ofrecida por el sistema de telediagnóstico.

## DISCUSIÓN

Nuestros resultados muestran que el sistema de telediagnóstico del MSPBS puede ser considerado como una herramienta muy prometedora para mejorar la calidad asistencial sanitaria del Paraguay.

La mejora en la atención y diagnóstico médico, en la reducción del tiempo promedio del diagnóstico, así como la extensión de servicios médicos a distancia en localidades en que estos no están disponibles, están en consonancia con lo referenciado por otros autores en otros países (5, 6). La implementación de este sistema en las tres áreas estudiadas aporta beneficios monetarios en relación a la reducción de los costos de la asistencia médica, los gastos de traslado de pacientes y del personal especializado. También, el sistema de telediagnóstico podría utilizarse como plan de contingencia para la asistencia médica en casos de catástrofes, epidemias, pandemias o ante cualquier evento con gran afluencia de pacientes (5).

El modelo en red del sistema de telediagnóstico permite una aplicación centralizada desde cualquier navegador web en forma remota, lo que lo hace accesible desde cualquier plataforma. La aplicación centralizada permite simplificar los procesos de mantenimiento y actualización del *software* operativo, aunque la utilización de ciertas herramientas para capturar y procesar las imágenes (y que son dependientes del sistema operativo) son limitados.

La incorporación del sistema de telediagnóstico en los centros asistenciales en salud implica una revisión y análisis de los procedimientos sistemáticos clásicos del servicio médico, debido a la innovación en la forma de registro, captación, transmisión y tratamiento de la información (imágenes y datos) desde el punto de vista científico, legal y ético (6, 7). Se ha visto, además, que para aprovechar los beneficios del telediagnóstico se tienen que garantizar los algoritmos de representación, transferencia y compactación de las informaciones generadas en el equipo de diagnóstico; la fiabilidad y seguridad de la transmisión (conectividad). La diferencia de efectividad diagnóstica entre ECG y los estudios de tomografía y ecografía se fundamentan sobre todo en el tamaño de los archivos de imágenes a transmitir y los factores enumerados en la sección de Materiales y métodos que influyen la variabilidad del telediagnóstico. Cabe mencionar también que todavía no existen regulaciones internacionales para el telediagnóstico que abarquen todos estos aspectos, a pesar de que ya existen algunos algoritmos de representación y transferencia de información que utilizan estándares de comunicación tales como el DICOM (8).

A la luz de la revisión de la literatura, y pesar de los esperanzadores resultados obtenidos con la telemedicina en países menos desarrollados (9), son todavía escasos los estudios que avalan de manera rotunda la efectividad y sostenibilidad de dicha tecnología (2, 4, 6, 10). La mayoría de los artículos analizados exponen la necesidad de metodologías más homogeneizadas y que, además, incluyan los costos totales de la implementación del sistema de telemedicina versus los costos sociales del traslado de los pacientes a lugares donde existe la posibilidad de realizar un diagnóstico “cara a cara” o de instalar en el punto remoto los recursos necesarios para hacer los estudios presenciales (11-39). En cualquier caso, consideramos que nuestro sistema de telediagnóstico muestra ventajas tales como la disminución de los tiempos de atención del paciente, diagnósticos más rápidos, mejora de la calidad del servicio con procedimientos padronizados, atención continuada para el diagnóstico remoto, posibilidad de interconsulta y envío del diagnóstico por internet al médico interviniente. En cuanto al “costo negativo” que se observó con la ecografía, se considera que se debe a que el sistema de teleecografía aún no ha alcanzado el valor umbral o punto de inflexión para garantizar su rentabilidad en relación a un diagnóstico “cara a cara”. Sin embargo, los diagnósticos que se han podido realizar gracias a este sistema, junto con el alto grado de satisfacción de los pacientes, hacen que se deba seguir explorando este sistema de telediagnóstico.

No obstante, y a pesar de los prometedores resultados en este estudio, antes de realizar su implementación masiva en los centros asistenciales de salud del interior del Paraguay consideramos que se deberá realizar un estudio más pormenorizado de los sistemas de salud, de los costos para su implementación, mantenimiento y sustentabilidad así como de la calidad diagnóstica del sistema acorde a las metodologías vigentes.

## Agradecimientos

Los autores desean agradecer a las instituciones que han apoyado el desarrollo del sistema de telediagnóstico, en particular a la Comisión Nacional de Telecomunicaciones (CONATEL); Telefonía Celular del Paraguay S.A. (TELECEL); Universidad del País Vasco, Bilbao, España; Organización Panamericana de la Salud (OPS) y a la Embajada de la República de China (Taiwán) en Paraguay. También se agradece el apoyo recibido de la Dirección General de Gabinete (DGG); Dirección de Tecnologías de la Información (DTIC); Dirección General de Administración y Finanzas (DGAF); y de la Dirección General de Recursos Humanos (DGRRHH), del Ministerio de Salud Pública y Bienestar Social de la República de Paraguay.

## Declaración

Los patrocinadores del estudio no participaron en el diseño del estudio, la recolección y análisis de datos, la decisión de publicar este trabajo y ni en la preparación del manuscrito. Las opiniones expresadas en este manuscrito son responsabilidad del autor y no reflejan necesariamente los criterios ni la política de la *RPSP/PAJPH* y/o de la OPS.
